# A phase I study of combination chemotherapy with gemcitabine and oral UFT for advanced non-small cell lung cancer

**DOI:** 10.1038/sj.bjc.6600337

**Published:** 2002-06-05

**Authors:** T Seto, K Yoh, H Asoh, H Yamamoto, H Semba, Y Ichinose

**Affiliations:** Division of Respiratory Diseases, Kumamoto Regional Medical Center, 5 Cho-me 16-10 Honjou, Kumamoto City 860 0811, Japan; Department of Thoracic Oncology, National Kyushu Cancer Center, 3-1-1 Notome Minami-Ku, Fukuoko City 811 1395, Japan; Department of Respiratory Medicine, Asou Iizuka Hospital, 3-83 Yoshiomado, Iizuko City 820 0018, Japan

**Keywords:** phase I study, non-small cell lung cancer, gemcitabine, UFT

## Abstract

A phase I study was carried out to determine the optimal dose and administration schedule for combined UFT plus gemcitabine therapy in patients with non-small cell lung cancer. Twenty-four patients (including 11 patients previously treated with cisplatin as the key drug) received oral UFT 400 mg m^−2^ on days 1 to 14 with intravenous infusions of gemcitabine (800 mg m^−2^ on days 8 and 15, or 900 mg m^−2^ on days 8 and 15, or 900 mg m^−2^ on days 1, 8 and 15). The most appropriate dosing option appeared to be 400 mg m^−2^ per day of oral UFT for 14 consecutive days with 900 mg m^−2^ gemcitabine on days 8 and 15. Eight of the 24 patients achieved partial response. The combination chemotherapy UFT and gemcitabine was well tolerated and may benefit patients with advanced non-small cell lung cancer. A multicentre phase II study using a 3-weekly regimen is in progress.

*British Journal of Cancer* (2002) **86**, 1701–1704. doi:10.1038/sj.bjc.6600337 www.bjcancer.com

© 2002 Cancer Research UK

## 

Although treatment of unresectable non-small cell lung cancer has begun to yield better results following the adoption of combination chemotherapy using cisplatin (CDDP) as the key drug, the median survival period of these patients still remains dismal at 6–10 months (Non-small cell lung cancer collaborative Group, 1995). Therefore, more efficacious treatment employing modalities that would entail minimal adverse effects, high efficacy rates and prolonged survival is needed.

Gemcitabine (GEM), a new anticancer drug structurally resembling cytosine arabinoside (Ara-C), has been shown to have high anti-tumour activity and minimal adverse effects (Hertel *et al*, 1988). Thus, it is reasonable to assume that gemcitabine could be better tolerated in older patients or patients with poor performance status (PS).

UFT, an oral antimetabolite compound composed of tegafur and uracil (1 : 4), also has mild adverse effects (Yamada *et al*, 1980). Earlier preclinical studies have demonstrated that it can inhibit tumour growth and prolong patients' lives through inhibition of tumour neovascularisation (Maehara *et al*, 1988; Ota *et al*, 1988).

Although the response rate of non-small cell lung cancer patients to UFT as a single agent is reported to be 6–8%(Keicho *et al*, 1986; Shimizu *et al*, 1986),combination chemotherapy using UFT plus cisplatin in those patients demonstrated a response rate of 35% and an extremely low incidence of adverse events(Ichinose *et al*, 1995).

Thus, both GEM and UFT are antimetabolites with minimal adverse effects that could be expected to provide a better quality of life (QOL). These two drugs inhibit DNA synthesis via different pathways, i.e., DNA chain termination and thymidylate synthase (TS) inhibition, respectively. We can therefore expect synergistic effects when they are used in combination. Based on this premise, a phase I study was carried out to determine the optimal dose and administration schedule for combined UFT+GEM therapy in patients with advanced non-small cell lung cancer.

## PATIENTS AND METHODS

The subjects were patients aged 80 years or younger with stage IIIB or IV non-small cell lung cancer (cytologically and histologically confirmed). They were not amenable to radical irradiation, with a PS score (ECOG) of 0–2 and a predicted survival of at least 3 months. The subjects were recruited at least 28 days after the previous treatment. The eligibility criteria in terms of organ functions were as follows: bone marrow function: total leukocyte count 4 000 μl^−1^ or higher, neutrophil count 2000 μl^−1^ or higher and platelet count 100 000 μl^−1^ or higher; liver functions: serum AST and ALT levels not more than twice the upper limit at the institution, serum total bilirubin level under the upper limit at the institution; renal functions: serum creatinine levels under the upper limit at the institution; pulmonary function: SpO_2_ 90% or greater.

The exclusion criteria were as follows: presence of pericardial effusion or pleural effusion necessitating drainage, interstitial pneumonia diagnosable on plain chest X-ray, history of serious cardiac dysfunction or episodes of ischaemia within the preceding 3 months, or symptomatic brain metastasis. Written consent was obtained from each patient.

### Drug administration

UFT was administered orally twice a day (before the morning and evening meals), at a fixed daily dose of 400 mg m^−2^, up to a maximum of 600 mg day^−1^. Capsules containing 100 mg UFT were used. When an odd number of capsules was used for a daily dose of, e.g., 500 mg, the dose was divided to 300 mg for the morning and 200 mg for the evening. GEM was dissolved in 20 ml of physiological saline, and then diluted further with physiological saline or 5% glucose to a volume of 250 ml. The GEM solution was administered by intravenous drip infusion over 30 min. On the day of GEM administration, a complete blood count was checked and the drug was administered only when the leukocyte count was 2000 μl^−1^ or higher and the platelet count was 70 000 μl^−1^ or higher. If these requirements were not met, the drug administration was postponed for a maximum of 4 days. The course of therapy was repeated at 28-day intervals. Recovery from bone marrow toxicity was confirmed before initiation of a further course.

### Dose escalation procedure and drug delivery schedule (Table 1)

**Table 1:**
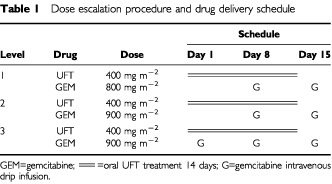
Dose escalation procedure and drug delivery schedule

In this phase I study, the UFT dose administration schedule was fixed at 400 mg m^−2^ per day on days 1–14. GEM was administered in incremental doses; 800 mg m^−2^ on days 8 and 15 in level one, 900 mg m^−2^ (the recommended dose for combined GEM therapy in Japan) on days 8 and 15 in level two, and 900 mg m^−2^ on days 1, 8 and 15 in level 3.

### Evaluation

Drug toxicity was evaluated after two courses of therapy. Dose-limiting toxicity (DLT) was defined as the presence of grade 4 leukopenia, neutropenia or thrombocytopenia according to WHO criteria for adverse reactions, fever of 38° or higher attributable to neutropenia, inability to fit the administration criteria for more than 5 days after the intended day of GEM administration, grade-3 or higher non-haematological toxicity, or unexpected serious adverse reactions. At least three patients were enrolled in level one, and three more patients were included when DLT occurred in not more than one patient. Then, the study was carried over to the next level when DLT occurred in not more than two patients. When DLT occurred in three or more patients, another six patients were included, and the dose causing DLT in 50% of the patients was determined and regarded as the maximum tolerated dose (MTD), the preceding dose being considered as the recommended dose.

## RESULTS

Twenty-four patients were enrolled between December 1999 and June 2000. Table 2

**Table 2:**
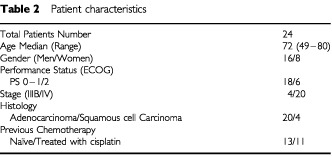
Patient characteristics

shows the patient characteristics. The 24 patients were comprised of 16 men and eight women, with a median age of 72 years (range, 49–80 years). Eleven patients had previously received chemotherapy with CDDP as the key drug. The number of patients included in each level is shown in Tables 3

**Table 3:**
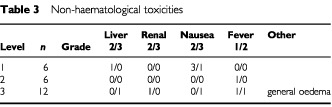
Non-haematological toxicities

and 4

**Table 4:**
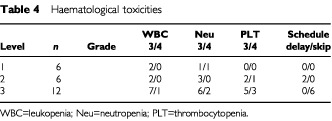
Haematological toxicities

. Each patient received 1–4 courses of therapy, with a median of three courses.

### Toxicity (Tables 3 and 4)

General oedema and capillary vasculitis in the lower legs occurred in one patient at level three, and this patient therefore received only one course of therapy. DLT occurred in one case (neutropenia) at level one. Three more patients were added, with DLT being noted in only one of the six total patients; the dose was then advanced to level two. At level two, thrombocytopenia was observed in one patient, and following the addition of another three patients, the frequency of thrombocytopenia observed was 1 out of 6. Although GEM administration on day 15 was postponed in two patients, the drug was administered within the subsequent 4 days. Twelve patients were studied in level three. Grade-3 hepatotoxicity and nausea was found in one patient. Leukopenia, neutropenia and thrombocytopenia occurred in one, two and three patients, respectively, and fever associated with leukopenia was noted in one patient. No central nerve system toxicity nor any lethargy was observed in this treatment. Since GEM administration scheduled on day 15 was not possible within the subsequent 4 days in six patients, the dose at this level was regarded as the MTD, and the level two dose was considered as the recommended dose.

### Response

Significant response was observed in eight out of the total of 24 patients. The overall response rate was 33% (95% confidence interval: 14 to 52%). The response rate among the chemo-naïve patients was 45% (5 out of 11), and that for the second-line use was 23% (3 out of 13).

## DISCUSSION

CDDP-based chemotherapy has been demonstrated to result in prolonged survival and improved QOL in patients with advanced non-small cell lung cancer (Non-small cell lung cancer collaborative Group, 1995). Several newer agents available for clinical use in the 1990's have been shown to provide even greater survival benefits than conventional drugs when used in combination with cisplatin (Bunn and Kelly, 1998). Among such new drugs, GEM is a new pyrimidine nucleoside analogue having antitumour activity with a unique mechanism of action (Hertel *et al*, 1988). Monotherapy with GEM has been shown to yield an efficacy rate of 20% or higher when used in patients with advanced non-small cell lung cancer (Gatzemeier *et al*, 1996; Abratt *et al*, 1994; Fukuoka *et al*, 1996). Its administration has also been shown to be associated with a low incidence of adverse reactions such as bone marrow suppression, nausea, vomiting and hair loss, suggesting that it might be well tolerated by elderly patients (Shepherd *et al*, 1997; Martin *et al*, 1997). Earlier studies have demonstrated that GEM could be combined with most other agents because of its unique mechanism of action as well as non-overlapping safety profile with these agents (Cortes-Funes *et al*, 1997). In particular, the efficacy rate of GEM administered in combination with CDDP has been reported to be 40–60% (Sandler and Ettinger, 1999). However, combination chemotherapy with CDDP could prove to be difficult in elderly patients and in those with a poor PS.

The oral antimetabolite UFT (a drug composed of tegafur and uracil mixed at the ratio of 1 : 4) has been shown in earlier studies to inhibit tumour growth and prolong survival through inhibition of tumour neovascularization. This drug also has minimal adverse effects and is presumed to be well tolerated in elderly patients. UFT has also been used in postoperative adjuvant chemotherapy against earlier stages of non-small cell lung cancer (Wada *et al*, 1996). The efficacy and survival rate of combination chemotherapy with UFT and CDDP are also reported to be favourable (Ichinose *et al*, 1995, 2000).

Chemotherapeutic regimens having low toxicity and utility in patients of advanced age or with poor PS are of major relevance in the treatment of non-small cell lung cancer in the future. In this regard, both GEM and UFT are antimetabolites with minimal adverse effects and offer promise for improvement in the QOL of these patients. They are expected to exert synergistic effects as they inhibit DNA synthesis via different pathways, i.e., DNA chain termination and TS inhibition, respectively. Synergism for GEM and 5-Fluorouracil (5-FU) combinations was found in colon cancer cell lines (Hagag *et al*, 1999)

The same concept combination chemotherapies with 5-FU and GEM have been reported as follows; a phase I study of folic acid plus 5-FU and GEM for solid tumour malignancies (Madajewicz *et al*, 2000), and a phase II study for advanced pancreatic cancer of 5-FU+GEM (Cascinu *et al*, 1999). According to these reports, the response rates for colon cancer and pancreas cancer were 28.5% and 3.7%, respectively. However, clinical benefits were obtained in 51% of patients with pancreas cancer, and this combination therapy is considered active treatment. Although other phase I studies of GEM in combination with UFT for treatment of gastrointestinal solid cancers have been conducted (Philip *et al*, 1999), the optimal dosing schedule has not yet been determined. A phase II study of UFT+GEM and leucovorin for advanced pancreatic carcinoma demonstrated a high response rate (16%) for this treatment (Feliu *et al*, 2000).

In the present study in patients with non-small cell lung cancer, GEM was administered in incremental doses of 800 mg m^−2^ on days 8 and 15 in level one and 900 mg m^−2^ (the recommended dose for combination therapy in Japan) on days 8 and 15 in level two. UFT was administered orally for 14 consecutive days at the fixed dose of 400 mg m^−2^ per day. GEM was administered in two schedules; on days 8 and 15 in levels one and two, and on days 1, 8 and 15 in level three. The administration on days 1, 8 and 15 in level three was associated with more severe haematologic toxicity, with the nadir usually occurring on day 15. Thus, GEM administration was not possible on day 15 in 50% of the patients at this level.

One important objective for the treatment of patients of advanced age or with poor PS in outpatient clinics is to administer a chemotherapeutic regimen on a weekly basis. Thus, severe bone marrow suppression and the inability to comply with the dosing schedule will be major deterrent factors. An *in vitro* study showed that anti-tumour cell activity of GEM was higher with previous 5-FU administration, compared to when 5-FU was given afterwards (Rauchwerger *et al*, 2000). Since oral UFT metabolises to make 5-FU, significantly higher levels of 5-FU were found on day 5 than day 1 in pharmacokinetic studies (Ho *et al*, 1998). In addition, the metabolic mechanism of UFT on day 1 would prevent synergism between GEM and UFT. Taking these into consideration, the level two regimen (oral UFT 400 mg m^−2^ per day for 14 consecutive days+GEM 900 mg m^−2^ on days 8 and 15) appears to be the most appropriate dosing option. Since the nadir of blood toxicity at level two was commonly found on days 16–18, a 3 weekly cycle might also be possible.

The overall response rate was observed in 33% of the patients in the current study. Among chemo-naive patients, the response rate was up to 45%, suggesting that the treatment could also be effective as primary treatment, even taking into consideration the incidence of toxicities. In addition, among the patients who had received previous chemotherapy with cisplatin, the response rate was 23%, suggesting the potential utility of the regimen as second-line chemotherapy. Based on the results of this phase I study, a multicentre cooperative group phase II study in chemo-naive patients with non-small cell lung cancer is now under way. This phase II study will be using a 3-weekly regimen consisting of UFT 400 mg m^−2^ per day for 14 days and GEM 900 mg m^−2^ on days 8 and 15. The dose may be elevated to 1000 mg m^−2^ in a second course if the incidences of bone marrow suppression are acceptable.
